# Multi‐tissue transcriptome‐wide association studies

**DOI:** 10.1002/gepi.22374

**Published:** 2020-12-28

**Authors:** Nastasiya F. Grinberg, Chris Wallace

**Affiliations:** ^1^ Department of Medicine, Jeffrey Cheah Biomedical Centre, Cambridge Biomedical Campus, Cambridge Institute of Therapeutic Immunology and Infectious Disease University of Cambridge Cambridge UK; ^2^ MRC Biostatistics Unit University of Cambridge Cambridge UK

**Keywords:** complex traits, gene expression, multi‐task learning, transcriptome‐wide association studies

## Abstract

A transcriptome‐wide association study (TWAS) attempts to identify disease associated genes by imputing gene expression into a genome‐wide association study (GWAS) using an expression quantitative trait loci (eQTL) data set and then testing for associations with a trait of interest. Regulatory processes may be shared across related tissues and one natural extension of TWAS is harnessing cross‐tissue correlation in gene expression to improve prediction accuracy. Here, we studied multi‐tissue extensions of lasso regression and random forests (RF), joint lasso and RF‐MTL (multi‐task learning RF), respectively. We found that, on our chosen eQTL data set, multi‐tissue methods were generally more accurate than their single‐tissue counterparts, with RF‐MTL performing the best. Simulations showed that these benefits generally translated into more associated genes identified, although highlighted that joint lasso had a tendency to erroneously identify genes in one tissue if there existed an eQTL signal for that gene in another. Applying the four methods to a type 1 diabetes GWAS, we found that multi‐tissue methods found more unique associated genes for most of the tissues considered. We conclude that multi‐tissue methods are competitive and, for some cell types, superior to single‐tissue approaches and hold much promise for TWAS studies.

## 1 INTRODUCTION

Genome‐wide association studies (GWAS) have been hugely successful over the last decade, transforming genetic association testing into a reproducible science (Kraft et al., 2009) and identifying tens of thousands of variants associated with more than a thousand traits (Buniello et al., 2019). However, lack of interpretability remains a criticism of GWAS (Visscher et al., 2012)—most disease‐associated variants lie in regulatory regions (Castel et al., 2018; Hindorff et al., 2009) but have not yet been convincingly linked to the genes they regulate. It has been noted that expression quantitative trait loci (eQTL) are over‐represented among trait‐associated single nucleotide polymorphisms (SNPs) uncovered by GWAS (Nica et al., 2010; Nicolae et al., 2010). This has motivated the development of different methods to link GWAS variants to genes by integrating GWAS and eQTL data sets (Guo et al., 2015; Marigorta et al., 2017; Zhu et al., 2016), and one promising approach, referred to as transcriptome‐wide association study (TWAS), is to use an eQTL data set to learn rules with which to impute gene expression in GWAS samples. Predicted gene expressions can then be used in place of genotypes within the standard GWAS framework, enabling gene‐based instead of variant‐based, case–control comparisons (Gamazon et al., 2015).

Previously proposed approaches for learning the imputation rules are based on regularized linear models (Fromer et al., 2016; Gamazon et al., 2015; Gusev et al., 2016; Mancuso et al., 2017), polygenic risk scores (Gamazon et al., 2015) and using the top SNP to predict expression levels (Gusev et al., 2016). However, the machine learning literature has shown that alternative approaches such as random forests (RF), which allow naturally for nonlinear and nonadditive effects, can produce more accurate predictions of complex traits (Michaelson et al., 2010; Sarkar et al., 2015; Xu et al., 2011). Recently, Fryett et al. (2020) conducted a comprehensive study comparing prediction accuracy of RF and a number of linear approaches in the TWAS situation. They found Bayesian sparse linear mixed model performed the best, followed by RF and the regularized regression methods lasso and elastic net. RF and regularized regressions have the additional advantages of being easily extensible to multi‐task learning framework, and so we chose to explore the degree to which incorporating information from multiple tissues could increase the power of TWAS.

A natural extension to TWAS is to take advantage of the fact that expression levels of a given gene in different cell types can be correlated by considering expression values across multiple cell types simultaneously in a multi‐task framework. This has been shown to improve multi‐trait predictions in yeast (Grinberg et al., 2019) and in applications to real and simulated data in marker‐assisted selection for several related traits (Calus & Veerkamp, 2011; Guo et al., 2014; Hayashi & Iwata, 2013) or populations (Chen et al., 2014). Multi‐trait approaches have also been used to analyse eQTL data sets (Flutre et al., 2013; Hu et al., 2019). While multi‐tissue extensions to TWAS have already been studied (Barbeira et al., 2019; Hu et al., 2019), to our knowledge, only linear approaches have been considered. We decided to evaluate performance of a nonlinear multi‐tissue approach. To do this, we adapted standard RF for this purpose and compared it to the joint lasso of Dondelinger and Mukherjee (2018), as well as to a selection of linear methods and RF trained on data from single tissue only.

## 2 METHODS

### 2.1 Accuracy of predicting gene expression

We first evaluated the utility of single‐task learning (STL) and multi‐task learning (MTL) models for predicting gene expression from genotype data using a train/test split of an eQTL data set from five immune cell types: B cells and (stimulated) monocytes from 430 individuals (Fairfax et al., 2012; Fairfax et al., 2014) (Table 1). In contrast to a classical (STL) predictive model which learns to predict just one target/output, an MTL model leverages similarities between targets of several regression problems by learning these targets simultaneously (Ben‐David & Schuller, 2003; Caruana, 1997). It is known that many eQTLs are active across multiple cell types (Aguet et al., 2017), so combining expression data sets of several related tissues can not only enhance predictive models' ability to uncover eQTL signals but also help to learn more about disease etiology when expression levels are imputed into a GWAS data set. In our context, this means building a gene expression prediction model using data for all available cell types. For an STL approach (building a separate regression model for each cell type), we trained RF (Breiman, 2001) and three regularized regressions: elastic net (Zou & Hastie, 2005), lasso (Tibshirani, 1994), and ridge (Hoerl & Kennard, 1970). For MTL we trained two models: joint lasso of Dondelinger and Mukherjee (2018) and an MTL version of RF (we call it RF‐MTL).

**Table 1:** Summary of the eQTL data set used in this study

Data set	Cell type	Samples	SNPs	Probes
Fairfax et al.	CD14^+^	413	588,141	47,230
	CD14^+^ LPS2	260		
	CD14^+^ LPS24	321		
	CD14^+^ IFN	366		
	B cell	284		47,231

*Note*: Expression data of Fairfax et al. (2014) includes B cells and monocytes, inactivated and activated—response to IFN and LPS after 2 h (LPS2) and 24 h (LPS24).

Abbreviations: IFN, interferon‐γ; LPS lipopolysaccharide; SNP, single nucleotide polymorphisms.

All expression values used in the STL models were standardized to have mean 0 and variance 1, individually for each cell type. For the MTL framework, for each eligible probe, we centered the expression values to have mean 0 (but did not standardize them) for each cell type individually.

For efficiency, the first step of our analysis was to filter probes with no genetic predictability. Even though standard univariate eQTL association analysis, by virtue of its linearity, does not show the full picture of relationships between SNPs and expression, it is fast and can help us to gauge the strength of genetic signal for each probe. For each probe, SNP markers within 1 Mbp of that probe (*cis*‐SNPs) were used to train a predictive model for each cell type. Only probes that have at least one cell type with a nominally associated *cis*‐SNP (*p* < 10^−7^; see Figure S1) were considered—4288 probes resulting in 21,440 probe–cell regressions. The cut‐off was chosen by examining the performance of the four predictive methods as a function of the *p* value threshold. The resulting Figure S1 indicates $10^{-7}$ to be a threshold around and above which ML methods start producing models with reasonably high $R^{2}$ (*R*‐squared; see Section 2.1.5) on a test set. Additionally, we excluded the HLA region (chr6:20–40 mbp). Probe positions, originally on build 38 (GRCh38), were lifted over to build 18 (NCBI Build 36.1) to match the genotypic data. Some probes could not be matched and were discarded. Hence, out of the original 47,231 probes, 25,005 survived the liftovers, of which 4288 passed the *p* value thresholding and were retained for analysis.

#### 2.1.1 Elastic net

Lasso and ridge regressions are penalized regressions differing by their use of an $L^{1}$ or $L^{2}$ penalty parameter, respectively, with elastic net being a mixture of the two. Lasso and ridge regression's only tuning parameter is the complexity parameter λ. The *cv.glmnet* function from the *R* package *glmnet* we used to fit these models chooses an appropriate sequence of λ values by fitting a “master” model using all the data and then finds an optimal value via internal 10‐fold cross‐validation. Elastic net, being a mixture of the lasso and ridge, has an additional parameter α∈[0,1] with α=1 corresponding to full lasso and α=0 to full ridge. Usually, the mixture parameter α is also tuned via cross‐validation, but often a fixed value is chosen, for example, Gamazon et al. (2015) simply use α=0.5.

#### 2.1.2 Joint lasso

Joint lasso is a type of linear regularized regression that handles multiple data sets simultaneously by estimating different regression coefficients for different tissues while encouraging coefficients of similar tissues to be closer. This is done by introducing an extra regularization term penalizing difference between coefficients of different subgroups ($L^{1}$ or $L^{2}$ penalty) depending on how similar these subgroups are with respect to a given dissimilarity measure.

We opted for the $L^{2}$ fusion version of the joint lasso as it requires less tuning compared to the $L^{1}$ fusion, and the original paper (Dondelinger & Mukherjee, 2018) reported a similar performance for both. We tuned the $L^{2}$ joint lasso for the fusion parameter γ (responsible for encouraging similar parameter estimates for similar sub‐data sets) via external fivefold cross‐validation and for the general penalty parameter λ via an in‐built *cv.glmnet* internal 10‐fold cross‐validation described above (i.e., within each iteration of the γ‐tuning CV, lasso would tune for λ via another cross‐validation routine). The sequence of γ values was taken as in the authors' example code (http://fhm-chicas-code.lancs.ac.uk/dondelin/SubgroupFusionPrediction). For any probe and two tissues i and j we set group‐specific penalty $\tau_{\mathrm{ij}}$ to $\rho_{\mathrm{ij}}/\max_{k\neq l}\{\rho_{\mathrm{kl}}\}$, where $\rho_{\mathrm{ij}}$ is the correlation between expression in tissues i and jin the Fairfax data set. However, in Dondelinger and Mukherjee (2018), authors remark that in practice using nonconstant (unity) τ's did not improve predictive performance of joint lasso. The joint lasso was implemented using the f user package.

#### 2.1.3 Random forest

RF is an ensemble tree‐based nonparametric method and requires relatively little tuning: the optimal number of trees is determined by assessing out of bag error as the forest is grown (we grew 500 trees which was sufficient for convergence) while it has been suggested that regulating depth of the trees (via minimum number of observations in terminal nodes) has limited benefits (Hastie et al., 2009; Segal, 2004). We incline to agree. We thus used the default parameter values: minimum number of observations in terminal notes at 5 (resulting in deep trees), and the number of random variables considered at each split at a 1/3 of all SNPs (parameters *min.node.size* and *mtry*, respectively). We used the *ranger* function in the *ranger R* package to fit RF.

#### 2.1.4 RF‐MTL

To implement multi‐trait prediction in RF, we simply concatenated expression values for the five tissue types into one long vector. Genotypic matrices were similarly stacked into one tall matrix and an id variable indicating which tissue/data set each point came from was added. Then, each individual could have up to five associated sample points, treated as independent observations. Since we are including approximately the same number of samples per individual, correlation between these sample points should not introduce imbalance/bias in the data and adversely affect the algorithm.

The id variable was available for splitting at each iteration of the RF algorithm *(always.split.variables = “id*” in the *ranger* function). This way, the size of the training data was increased and subsets corresponding to different tissues could be separated or pulled together (via tree branching) depending on their dissimilarity or similarity, respectively. For genes with highly correlated expression values across different cell types, the id variable tends to be less important (i.e., not used for splits), the whole data set being treated as homogeneous. For genes exhibiting less or no correlation across different cell types, the id variable would split samples into different subsets forcing them into separate end nodes.

For RF‐MTL, the pooled approach should cater for situations when the underlying sub‐data sets have a varying degree of similarity. Pooling completely homogeneous (or even identical) datasets, should not adversely affect performance as the tissue id variable, although available as a splitting variable at every split, does not have to be used if it does not help reduce residual variance for a given tree. Strong differences between sub‐groups, on the other hand, should be handled by the use of the tissue id variable at various splits, effectively separating samples into homogeneous subsets. Thus arguing, we of course assume that similarities/dissimilarities between different subgroups are reflected in similarities/dissimilarities of their respective distributions over features.

#### 2.1.5 Evaluation of methods

Models were trained on a training set and evaluated on a test set, comprising roughly 70% and 30% of the data, respectively. To avoid information leaking in the MTL set‐up, all samples from the same individual were designated to either the training or the test set.

We used $R^{2}$ as a measure of predictive accuracy of different models. For a predictive model f, $R^{2}$ is informally known as the “proportion of the variance explained” by f and is defined as:

$$1-\frac{{\;}_{\sum }^{i}{\left(y_{i}-f(x_{i})\right)}^{2}}{{\;}_{\sum }^{i}{\left(y_{i}-\bar{y}\right)}^{2}}\approx 1-\frac{\mathrm{MSE}}{{\hat{\sigma }}^{2}},$$

where ${f(x}_{i})$ is prediction at point $x_{i}$, $\bar{y}$ is sample mean of outcome y, ${\hat{\sigma }}^{2}$ is *y*'s sample variance and MSE is mean square error. Note that the above fraction is a measure of how well f does compared to the “base” constant model $g(x_{i})=\bar{y}$, ∀i. One would expect a “good” model to have small MSE compared to ${\hat{\sigma }}^{2}$, and hence larger $R^{2}$. Conversely, a “bad” model would have a larger MSE and smaller $R^{2}$, with a truly hopeless model performing en par with a constant mean predictive function. Note also that, while the phrase “proportion of variance explained” suggests a value of $R^{2}$ in the interval [0,1], in reality the definition above does not put any such restriction on $R^{2}$. Indeed, a heavily overfitting model, or that trained and tested on data coming from vastly different distributions, can produce large negative $R^{2}$ values.

For two methods, $m_{1}$ and $m_{2}$, trained and validated on the same data sets with respective *R* ^2^ ${\;}^{R}$,$R^{2}$ $R_{m_{1}}^{2}$ and $R_{m_{2}}^{2}$, we say that $m_{1}$ *has an advantage over* $m_{2}$ if $R_{m_{1}}^{2}>0$ and $R_{m_{1}}^{2}>R_{m_{2}}^{2}$. This advantage is quantified by $R_{m_{1}}^{2}\underset{\max}{-}\{0,R_{m_{2}}^{2}\}$. The *average advantage* of $m_{1}$ over $m_{2}$ is calculated over a set of regression problems to which both methods are applied and $m_{1}$ has an advantage over $m_{2}$. In essence, the average advantage indicates by how much on average method $m_{1}$ is more accurate than method $m_{2}$ for problems, where $m_{1}$ does outperform $m_{2}$.

### 2.2 A simulation study of the utility of each prediction method for TWAS

We assessed the performance of each eQTL prediction method for TWAS in a simulation framework. Within each simulation, we simulated separate eQTL and GWAS data sets. For each data set, we first sampled independently 400 pairs of haplotypes from the 1000 Genomes EUR subset to generate genotype data, and sampled causal variants independently from among the SNPs.

For the eQTL (GWAS) data sets, 5 (1) quantitative traits were simulated, respectively, as Gaussian variables with variance 1 and mean $\sum_{i}^{\;}\beta_{ij}G_{i}$, where i indexes causal variants, j indexes traits, and $\beta_{\mathrm{ij}}$ is the effect size of variant i on trait j and $G_{i}$ the genotype vector at variant i. To avoid too many simulations with small beta and nonsignificant effects, $\beta_{i}$ was sampled as the maximum of five Gaussians with variance 0.04. The first expression trait was assigned as the trait to be tested via TWAS, and the remainder as additional “background” expression traits. Each expression trait was regressed against all SNPs, and the simulation retained if the minimum *p* value over all SNPs and expression traits was less than $10^{-7}$.

We conducted TWAS with each of the four methods described above, following the steps:

1. learn a predictive model in the eQTL data set
2. predict values for the first expression trait into the GWAS data set
3. test association between the GWAS trait and the predicted expression trait in the GWAS data set using linear regression

and retained the *p* value from this test.

The aim of TWAS is to associate genes and diseases. Although association can be thought necessary for causation, it is not sufficient (Wainberg et al., 2019). We use colocalisation analysis to determine whether, for a predicted gene expression with significant association to a GWAS trait, the same genetic signal underlies the eQTL and a trait‐association, or whether two (or more) distinct signals exist in linkage disequilibrium (LD). The colocalisation test is expected to preferentially filter out significant TWAS results that result from an eQTL variant distinct from, but in LD with, a GWAS causal variant. We do this via testing for proportionality of SNP regression coefficients for the two traits in question (Wallace, 2013). This alternative framing of the null hypothesis differs from the more widely known enumeration method for colocalisation (Giambartolomei et al., 2014) (where the null hypothesis is no association for either trait) and is a more natural way to approach this question once a joint association has been found. Our approach is thus related to the two‐stage HEIDI/SMR approach proposed by Zhu et al. (2016). Colocalisation validation was also used in Fromer et al. (2016) and Marigorta et al. (2017). However, recently other methods of validating/fine‐mapping TWAS signals have been proposed—Mancuso et al. (2019), for example, extend probabilistic SNP‐level fine‐mapping approaches to create credible sets of genes which explain a given TWAS signal with a given probability.

To reduce the degrees of freedom of the test, proportionality testing works by first finding principal components (PCs) of the genotype matrix accounting for the majority of the variation (usually 80%), and then regressing the two traits on these PCs. Finally, a null hypothesis that the two sets of coefficients are proportional (there is a colocalisation) is tested (at 0.05 significance level). To reduce the number of PCs used, we only used SNPs with GWAS or eQTL *p* < 10^−4^ and all the SNPs in their LD pockets ($r^{2}>0.2$ with selected SNPs), and selected the PCs accounting for at least 80% of the variation, or the first six PCs, whichever number is the smallest.

We ran proportional filtering on each simulated data set, and stored its *p* value, $p_{f}$. We assessed TWAS performance according to the proportion of simulations that gave a TWAS *p* < .05, before and after filtering at *p_f_* < .05.

### 2.3 TWAS study of type 1 diabetes (T1D)

To compare performance of the predictive methods in a real‐world data set, we retrained the models on the whole eQTL data (as opposed to 70% training set) and used them to impute (predict) gene expression into a large T1D GWAS cohort (Barrett et al., 2009); see Table S1. For some probes no SNPs are shared between the GWAS and the eQTL data set, so out of the initial 4288 probes, we are left with 4103. GWAS genotypes are then fed into the trained models to obtain *predicted* gene expression for GWAS individuals. Note that for the joint lasso and the RF‐MTL methods, only one model is needed for each probe, rather than one model for each probe/cell pair. To obtain predictions for a particular cell type, genotypic data was fed to the model together with the id variable indicating which tissue type we would like a prediction for. We then tested for association between the imputed expression levels and the disease status of the individuals in the GWAS data set, to see which probes/genes are differentially expressed. We used the Cochran‐Armitage test (Clayton & Hills, 2013) with Mantel adjustment to accommodate stratification in the GWAS design which involved two genotyping chips (Table S1). Note that the same number of tests of association between predicted gene expression and T1D status was performed for STL and MTL methods (i.e., one for each method/cell pair) despite fitting fewer predictive models for MTL methods. To account for multiple testing, the resulting *p* values were adjusted using the Benjamini–Hochberg (Benjamini & Hochberg, 1995) method (separately for each method and cell type). For the two lasso methods the total number of fitted models, as opposed to just the non‐null ones (a null model is one returning no nonzero coefficients), were used for the *p* values adjustment. This was done to avoid giving lasso and joint lasso an unfair advantage over the two forest models. We define a *TWAS‐significant* association (or hit/gene) as a cell‐probe‐method triplet for which predicted expression has a significant fold change, that is, an FDR‐adjusted Cochran‐Armitage test *p* < .05.

We then passed all the TWAS‐significant hits through the proportionality filter, described above. Thirteen out of 224 TWAS‐significant probe–cell pairs (corresponding to six probes) did not have enough SNPs with sufficiently small *p* values for the colocalisation procedure to be applied and were dropped. We call TWAS‐significant hits passing the proportionality filter *SP‐hits* (significant and proportional).

## 3 RESULTS

### 3.1 RFs allow improved predictions of gene expression in single tissues

We started by assessing single‐tissue models. Among the linear methods, ridge regression strictly underperformed compared to lasso and elastic net which performed similarly to each other, with lasso slightly preferred (Figure S2a), suggesting that eQTL prediction benefits from sparsity introduced by the elastic net and lasso regression. Moreover, once sparsity is introduced, varying the mixing parameter hardly affected performance of elastic net (Figure S2b), which agrees with the results of Fryett et al. (2020) who also found sparsity to be beneficial. We, therefore, dropped ridge regression and elastic net from further analysis.

**Figure 1:**
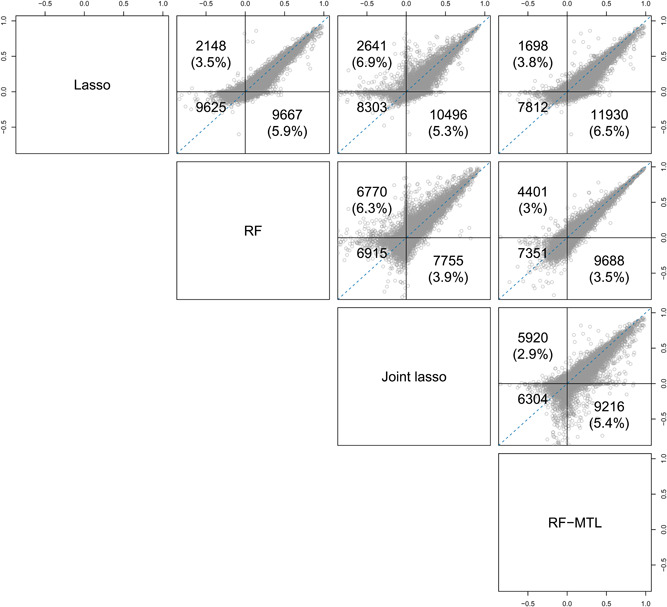
Pairwise comparison of performance of the MTL and STL expression prediction methods—$R^{2}$ on a test set. Each point represents a probe–cell pair. Points above the blue line show increased performance for the method to the left of each plot, while points below the blue line show increased performance for the method underneath the plot. The three numbers represent, clockwise: points with positive $R^{2}$ above x=y line for the x‐axis method, points with positive $R^{2}$ below the line for the y‐axis method, points with negative $R^{2}$ for both methods. Numbers in brackets represent the corresponding advantage of one method over the other, in terms of $R^{2}$ (for this calculation negative $R^{2}$ are taken to be 0). For example, comparing lasso and RF, lasso outperformed RF in 2148 regressions with an advantage of 3.5%, while RF outperformed lasso in 9667 with an advantage of 5.9%, and for 9625 probe–cell pairs neither method achieved a positive $R^{2}$. MTL, multi‐task learning; RF, random forest; STL, single‐task learning

RF outperformed lasso in the overwhelming majority of regressions with mean advantage (see Section 2) of RF over lasso of 5.9%, compared to 3.5% of mean advantage of lasso over RF (Figure 1). Moreover, for 1927 out of 11,814 probe/cell pairs with any signal, RF beats lasso by more than 10%. Points in the top left quadrant of the RF‐lasso graph correspond to regressions where RF has positive $R^{2}$ but lasso fails to produce a useful model (negative $R^{2}$).

### 3.2 Combining information from multiple cell types using multi‐task learning

We compared MTL extensions of lasso and RF to each other and to the reference models fitted on individual tissue types (STL). We considered the same 4288 probes for which at least one cell type has a nominally associated *cis*‐SNP *p* value ($p<10^{-7}$), resulting in the same number of regressions (each able to predict expression for five cell types).

Joint lasso outperforms standard lasso in the absolute majority of cases (Figure 1). However, joint lasso significantly underperforms in a handful of cases, against lasso as well as RF and RF‐MTL. RF‐MTL and RF are relatively evenly matched, although RF‐MTL performs slightly better in more regressions. RF‐MTL outperforms joint lasso substantially more often than the other way around (9161 and 5918 regressions, respectively) and tends to have a larger advantage (5.4% compared to 2.9% on average). Overall, RF‐MTL, on average, is the most accurate predictive model for our eQTL data set. Additionally, only one regression has to be fitted to cater for all cell types instead of one per cell type.

### 3.3 Simulation‐based comparison of learning methods for TWAS

**Figure 2:**
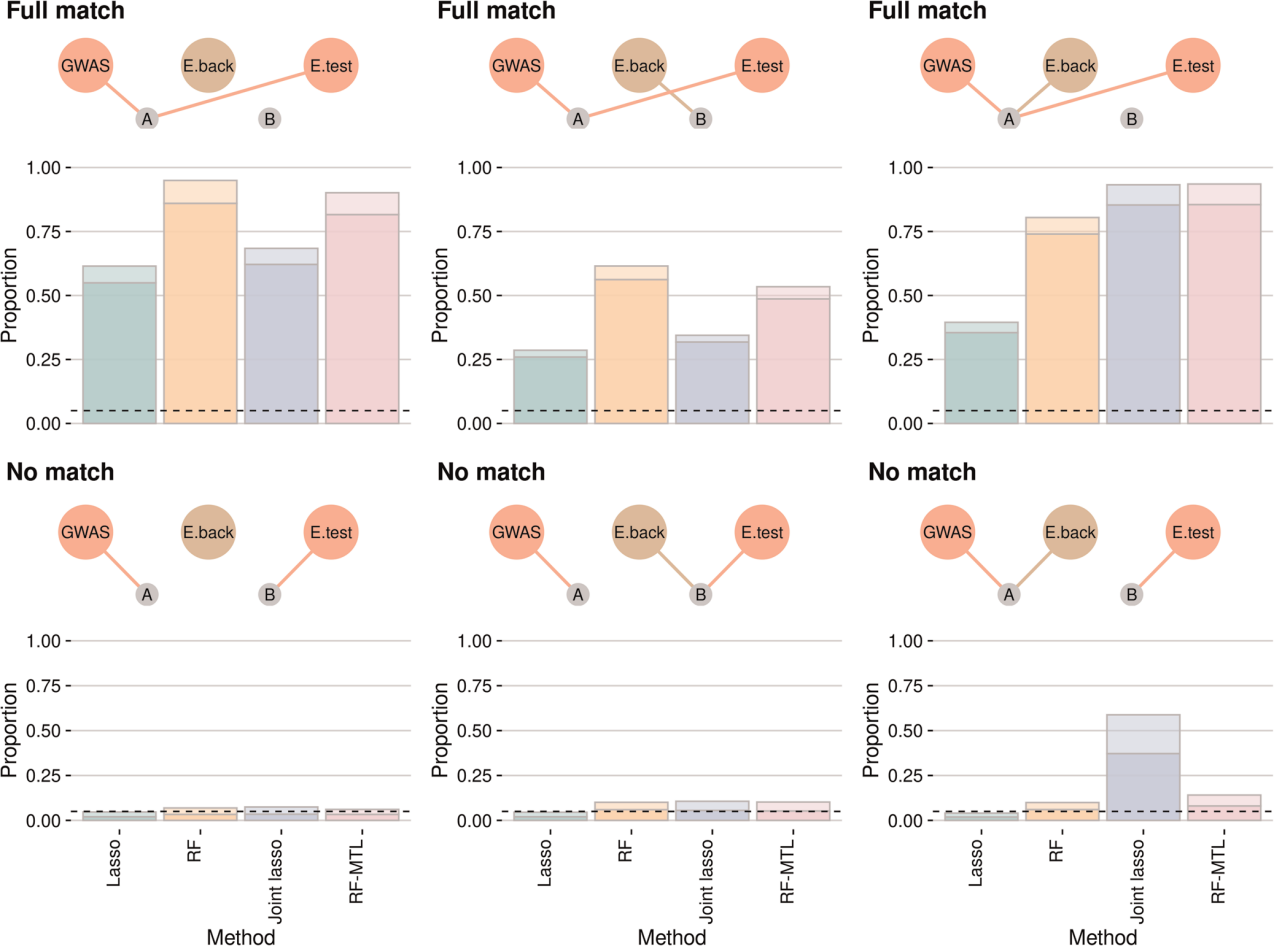
Power of different methods to detect TWAS association. In the top row, the GWAS and test eQTL traits share causal variant A, while the causal variant for the four background eQTL traits varies (left‐right) from none, to B to A. The bottom row is the same, except the GWAS and eQTL‐test causal variants are different. The total shaded column height is the proportion of TWAS tests that pass *p* <.05, with lighter shading used to indicate the proportion of tests which would be filtered out proportionality testing at *p* <.05. The horizontal dotted line is at y=0.05, the proportion of false‐positives expected in a well controlled testing procedure in the bottom row. eQTL, expression quantitative trait loci; GWAS, genome‐wide association study; TWAS, transcriptome‐wide association study

To assess the performance of the four methods as part of the complete two‐stage TWAS procedure, we simulated GWAS‐trait and gene expression data for five cell types under several genetic causal scenarios. Generally, when colocalised GWAS and eQTL signals were simulated, multi‐trait methods outperformed single‐trait methods when eQTL variants were shared between the test and background expression traits, and single‐trait methods performed slightly better when there was no sharing, though the difference was more pronounced in the former versus the latter (Figure 2, top panels). However, the situation was very different when background expression traits shared a variant with the GWAS but the test expression trait did not. Here, we might expect an increase in false‐positives due to occasional LD between GWAS‐trait variants and test‐expression‐trait variants, possibly explaining the higher false‐positive rate for unfiltered RF‐MTL compared to RF (0.14 and 0.10, respectively). However, joint lasso performed particularly poorly in this scenario, with a false positive rate (at a 0.05 threshold) of 0.58 compared to 0.040 for single‐task lasso. Testing proportionality was successful at preferentially filtering out false‐positives, reducing type 1 error rates to at or below their nominal value with the exception of the joint lasso case, where the false‐positive rate was only reduced to 0.37. Proportionality filtering also removed between 7.5% and 10.5% of true‐positives, fairly evenly across methods.

Overall, this suggests that the benefits of RF‐MTL over RF, and of RF over lasso for prediction transfer to TWAS. On the other hand, they warn that joint lasso may have a high false‐positive rate if interpreted in a tissue‐specific manner. A more detailed comparison of single‐task RF and lasso showed that the effects of regularization on lasso caused systematic over‐estimation of the causal effect of the expression on the GWAS trait with a lasso (Figure S5).

### 3.4 Forty‐six genes show predicted differential expression in T1D

**Figure 3:**
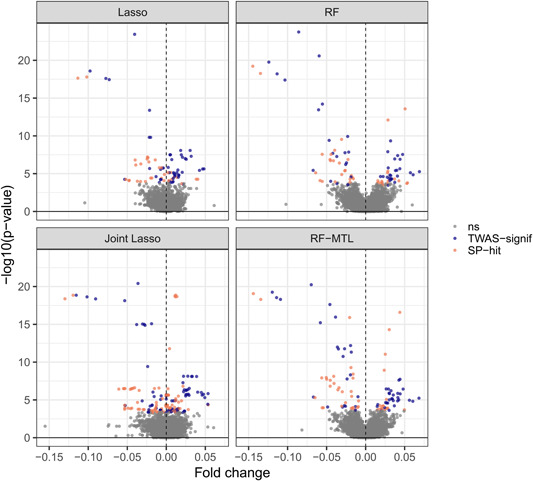
Volcano plots for testing association between the predicted gene expression and the T1D status. Grey points are not TWAS‐significant, blue points are TWAS—but not passing proportionality test, and orange points are both TWAS—and proportionality‐significant (SP‐hits). RF, random forest; TWAS, transcriptome‐wide association study

**Figure 4:**
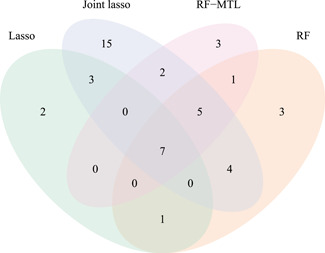
Unique TWAS‐significant hits passing proportionality filtering, by method: lasso (13), RF (21), joint lasso (36), and RF‐MTL (18). RF, random forest; MTL, multi‐task learning; TWAS, transcriptome‐wide association study

**Figure 5:**
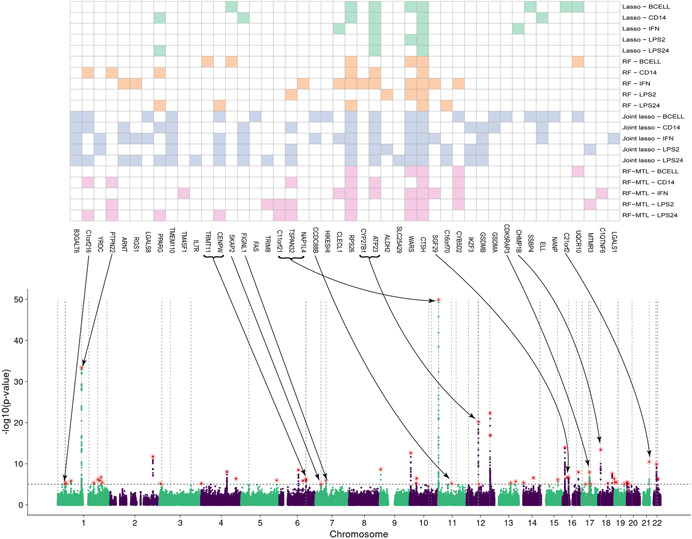
A heatmap of genes identified by the four methods after proportionality filtering (top), integrated with a Manhattan plot of type 1 diabetes GWAS. Arrows point to GWAS peaks (red stars) in the vicinity of which (1 Mbp either way) a gene (or several genes, grouped by a bracket) lies. Vertical dotted lines indicate positions of genes; horizontal dotted line is at $-\log_{10}p=5$, corresponding to a GWAS significant level of $10^{-5}$; green and purple colors in the Manhattan plot designate alternating chromosomes. Note that the genes in the heatmap are ordered according to their positions, so for any two genes (or groups of genes) an arrow from a leftmost one would point to a peak left of the peak pointed at by the rightmost gene. Any intersection between the arrows is due to the fact that they might point to peaks of vastly different heights. GWAS, genome‐wide association study

In our application to T1D, 62 distinct TWAS‐significant genes (adjusted *p* < .05, see Section 2) were identified by at least one of the four methods with joint lasso identifying the most (see Table 2, column 4). Filtering for proportionality left 46 distinct genes (Table 2 and Figure 3; see Supporting Information for a full list). These are SP‐hits (significant and proportional, see Section 2). There is a substantial overlap between the four methods but each also identified unique hits not discovered by the others (Figure 4 and S6). RF finds an equal or greater number of unique SP‐hits than lasso in all but one cell type. Likewise, RF‐MTL finds at least as many or more unique SP‐hits than single‐tissue RF in three out of five tissue types. Joint lasso identifies the most TWAS‐significant and SP‐genes for each cell type but these genes tend to be significant for three and more tissue types. Top of Figure 5 shows a heatmap of SP‐genes (columns) for the four methods for each cell type (rows) and not only the joint lasso portion of the heatmap is more populated than the ones corresponding to the other methods, but we also notice multiple full vertical lines designating instances when a gene is significant in all the cell types (see Section 4). Finally, we note that out of 46 unique SP‐hits 16 lie in the vicinity (within 1 Mbp) of a T1D GWAS SNP (*p* < 10^−5^); see Figure 5 for identity and location of these genes. Many of the other 30 relate to regions that did not achieve nominal significance ($p<10^{-5}$) in this study, have been robustly associated with T1D in other studies, including *CLECL1* (Burton et al., 2007), *RGS1* (Smyth et al., 2008), *IKZF3* (Burren et al., 2014), *IL7R* (Todd et al., 2007), and *CTSH* (Cooper et al., 2008).

**Table 2:** Table of results of the TWAS analysis

Method	Cell	*N*	TWAS‐significant (unique)	SP‐hits (unique)
Lasso	BCELL	1155	25 (18)	10 (8)
RF	BCELL	4103	17 (10)	8 (6)
Joint lasso	BCELL	3886	44 (36)	22 (19)
RF‐MTL	BCELL	4103	17 (11)	6 (5)
Lasso	CD14	1962	14 (11)	8 (6)
RF	CD14	4103	15 (12)	8 (6)
Joint lasso	CD14	3485	32 (26)	19 (15)
RF‐MTL	CD14	4103	20 (15)	10 (7)
Lasso	IFN	1919	14 (10)	5 (4)
RF	IFN	4103	30 (24)	13 (11)
Joint lasso	IFN	3494	40 (32)	22 (18)
RF‐MTL	IFN	4103	23 (18)	10 (9)
Lasso	LPS2	1317	10 (8)	5 (3)
RF	LPS2	4103	11 (10)	5 (4)
Joint lasso	LPS2	3762	33 (29)	17 (15)
RF‐MTL	LPS2	4103	21 (16)	11 (9)
Lasso	LPS24	1525	16 (13)	4 (3)
RF	LPS24	4103	13 (11)	6 (5)
Joint lasso	LPS24	3645	35 (31)	21 (19)
RF‐MTL	LPS24	4103	19 (15)	10 (9)
Total (unique)			449 (62)	220 (46)

*Note*: Non‐null regressions (N) refer to the expression prediction models taken through to the GWAS imputation state, that is, lasso and joint lasso models which identify no useful SNPs, and hence offer only constant predictions, are dropped. TWAS‐significant hits refer to predicted gene expressions passing the Cochran‐Armitage test (5% with Benjamini‐Hochberg adjustment) for differential expression in T1D. Finally, last column is the number of TWAS‐significant hits passing the proportionality filter (at 5%)—SP‐hits.

Abbreviations: IFN, interferon‐γ; LPS, lipopolysaccharide; MTL, multi‐task learning; RF, random forest; SNP, single nucleotide polymorphisms; TWAS, transcriptome‐wide association study.

As the complete list of true T1D genes is not known, we decided to compare the results from the different methods by passing the gene list to the target validation web analysis platform (https://www.targetvalidation.org) and searching for associated diseases, excluding genetic association data from the data types included to avoid circular reasoning. We ranked the diseases listed according to their relevance *p* value, and found that the RF‐based gene lists ranked more obviously T1D‐related diseases higher than lasso‐based gene lists (Table S2). Indeed, the term “type I diabetes mellitus” was the second ranked for RF and the third ranked for RF‐MTL, but only the 19th for lasso (19th) and 45th for joint lasso (45th), supporting that RF‐based TWAS was identifying disease‐relevant genes identified by methods independent from genetic association data.

## 4 DISCUSSION

The current ubiquity of linear methods in eQTL studies reflects both the speed and flexibility of these methods, but also the prevailing dogma that gene expression is influenced additively over variants and over alleles at those variants. This expectation reflects the lack of evidence from human studies directly targeting epistatic effects (Brown et al., 2014; Hemani et al., 2014; Powell et al., 2013). However, this lack of evidence could also reflect a lack of power (Timpson et al., 2018). While exploiting RF was not unreservedly a more powerful method for TWAS, the fact the RF predictions were generally better than those from lasso suggests that nonadditive effects make an important contribution in gene expression. Such nonlinearity has been detected in detailed molecular studies of individual genes (Baeza‐Centurion et al., 2019), and in large‐scale studies of model organisms (Celaj et al., 2020). It also motivates wider development and adoption of methods that can exploit nonadditivity where it exists, even in samples insufficiently large for nonadditivity to be robustly detected.

It is important to understand the reasons behind differences in performance of the four methods, both in terms of predictive accuracy and the number of TWAS‐significant hits discovered. Both tree‐based methods outperformed their linear counterparts on average, with the RF‐MTL being the most accurate overall. Clearly, while the lasso methods are competitive, RF‐based methods successfully exploit the supposed nonlinear relationships in the data. For T1D, however, this predictive advantage did not translate into more TWAS‐significant hits consistently across different tissue types. The reason for this may lie in the fundamental differences in the properties of the two models. Lasso (and so, joint lasso) produces biased solutions (unlike standard linear regression) with the resulting coefficients biased towards zero, accepting this cost to generate predictions with lower variance. Random forest, on the other hand, produces a low‐bias model but higher variance predictions (see Figures S3 and S4). As a consequence, even lasso predictions resulting in very small fold changes can lead to TWAS‐significant hits through incorporating few (sometimes just one) but important SNPs in predictive models (i.e., highly biased but low variance predictions). This can be seen most clearly comparing the shape of the volcano plots (Figure 3), where the expected dip in the middle is not evident in lasso. Overall lower variance of RF‐MTL predictions but similar size of predicted fold change, as compared to RF, might also explain why RF‐MTL does better in the TWAS framework.

Multi‐tissue methods demonstrated their applicability to TWAS both in terms of accuracy of models constructed on the eQTL data set and the number of unique TWAS‐significant genes and SP‐genes associated to TID identified. Indeed, Hu et al. (2019) found that their multi‐tissue method UTMOST outperformed single‐tissue elastic net, PrediXcan of Gamazon et al. (2015), in both stages of the TWAS framework. Like joint lasso, the UTMOST predictive model is a type of regularized regression with several penalty terms in addition to the standard least‐squares loss. The two penalties used in UTMOST are: $L^{1}$ for effect sizes within each tissue for variable selection and effect size shrinkage, and $L^{2}$ grouped lasso penalty for effect sizes across tissues to encourage cross‐tissue eQTLs. RF‐MTL, on the other hand, uses expression data from different tissues in a flexible nonparametric manner, exploiting similarities where they exist.

Various other MTL approaches exist and there is space for exploring their applicability to TWAS in future work. An ensemble tree method of gradient boosting machines (GBM; Friedman, 2001) can for example, be adapted for this purpose in the same way as RF. Random effects models (Balasubramanian et al., 2013) (once again a linear sparse model) and neural networks have also been adapted to multi‐task learning. The latter is an especially intriguing alternative, with a choice of a soft parameter sharing (Duong et al., 2015; Yang & Hospedales, 2017) (each task has its own hidden layers and parameters with the distance between parameters regularized) and hard parameter sharing (Caruana, 1993) (each task has individual hidden layers as well as layers shared between all the tasks).

The effects of regulatory variation have been shown to vary between cell types (Fairfax et al., 2012), and cell type‐specific chromatin accessibility has been used to associate multiple immune cell types to autoimmune disease GWAS (Farh et al., 2015). Hence, for a given disease, it is important not only to identify potential genes of interest but also the relevant tissue (s). Simulations showed that the two multi‐tissue methods we studied tend to “overborrow” information across tissues, that is, find significant hits for tissues without one if there is a real signal in another tissue. This was mostly a problem suffered by joint lasso and, to a much smaller extent, by RF‐MTL. It is harder to identify this behavior in real data. However, the number of TWAS‐significant hits identified by joint lasso in our T1D data and the fact that it was much more likely to find signal in three or more tissues for a given gene than the other methods, suggests similar behavior. Moreover, calculated standard deviation of predicted fold change for different cell types for each probe (for lasso methods, for probes with at least three cell types with non‐null predictions) reveal that joint lasso has the least variation in fold change predictions between different tissue types (see Figure S7). Hence, whilst outperforming single‐tissue lasso on average in terms of prediction accuracy, joint lasso seems to suffer from lower prediction specificity and, as a result, a higher rate of false‐positive TWAS‐hits in the TWAS framework.

Colocalisation testing is an important part of the TWAS framework and provides an in silico validation step for the identified associations. However, we note that associated genes filtered for lack of proportionality would be expected to be differentially expressed in healthy individuals at different risks of disease (those who carry greater or lesser burdens of disease‐predisposing variants). Thus, we might expect them to also be differentially expressed between cases and controls in a hypothetical study in which expression is measured directly. Therefore, we suggest such genes might be considered as biomarkers rather than red herrings. Even TWAS‐hits passing colocalisation tests can be validated only through practical lab‐based experiments.

In this study, we demonstrated the applicability of nonlinear and multi‐tissue methods in the TWAS framework. Both real data and simulation studies showed, in particular, that RF is at least as competitive and, for some tissue types, superior to lasso. Similarly, RF‐MTL is superior to RF for some tissue combinations, whilst joint lasso identifies more unique SP‐hits than lasso for all the tissue types. Our results highlight the potential to exploit multiple tissue‐eQTL studies in TWAS but we expect this to be most useful when tissues are closely related, so that information may be legitimately borrowed between tissues.

## SOFTWARE

All analysis was done in *R* using *glmnet* for lasso and elastic net, *ranger* for RF and RF‐MTL, and *fuser* and bespoke helper functions https://github.com/stas-g/fuser_helper for the joint lasso. coloc package was used for the post‐hoc colocalisation analysis. All simulation code is available from https://github.com/chr1swallace/twas-sims.

## Supporting information

Supporting information.

Supporting information.

Supporting information.

Supporting information.

Supporting information.

Supporting information.

Supporting information.

Supporting information.

Supporting information.

Supporting information.

## Data Availability

Data used in this study can be obtained from its original sources. Gene expression data is available through ArrayExpress: http://www.ebi.ac.uk/arrayexpress/experiments/E-MTAB-945 and http://www.ebi.ac.uk/arrayexpress/experiments/E-MTAB-2232. Genotyping data for the eQTL data set is available from the European Genome‐Phenome Archive: http://www.ebi.ac.uk/ega/EGAD00010000144 and http://www.ebi.ac.uk/ega/EGAD00010000520. 2000 T1D samples were genotyped as part of the WTCCC (and controls) ‐ data access is described https://www.wtccc.org.uk/info/access_to_data_samples.html. An additional 4000 cases were genotyped by the T1DGC, available at https://www.ncbi.nlm.nih.gov/projects/gap/cgi-bin/study.cgi?study_id=phs000180.v3.p2.
