# Supplementary figures and images for: Multi‐tissue transcriptome‐wide association studies
Source: Genet Epidemiol. 2020 Dec 28;45(3):324–37. doi: 10.1002/gepi.22374 (PMC8048510; doi:10.1002/gepi.22374)

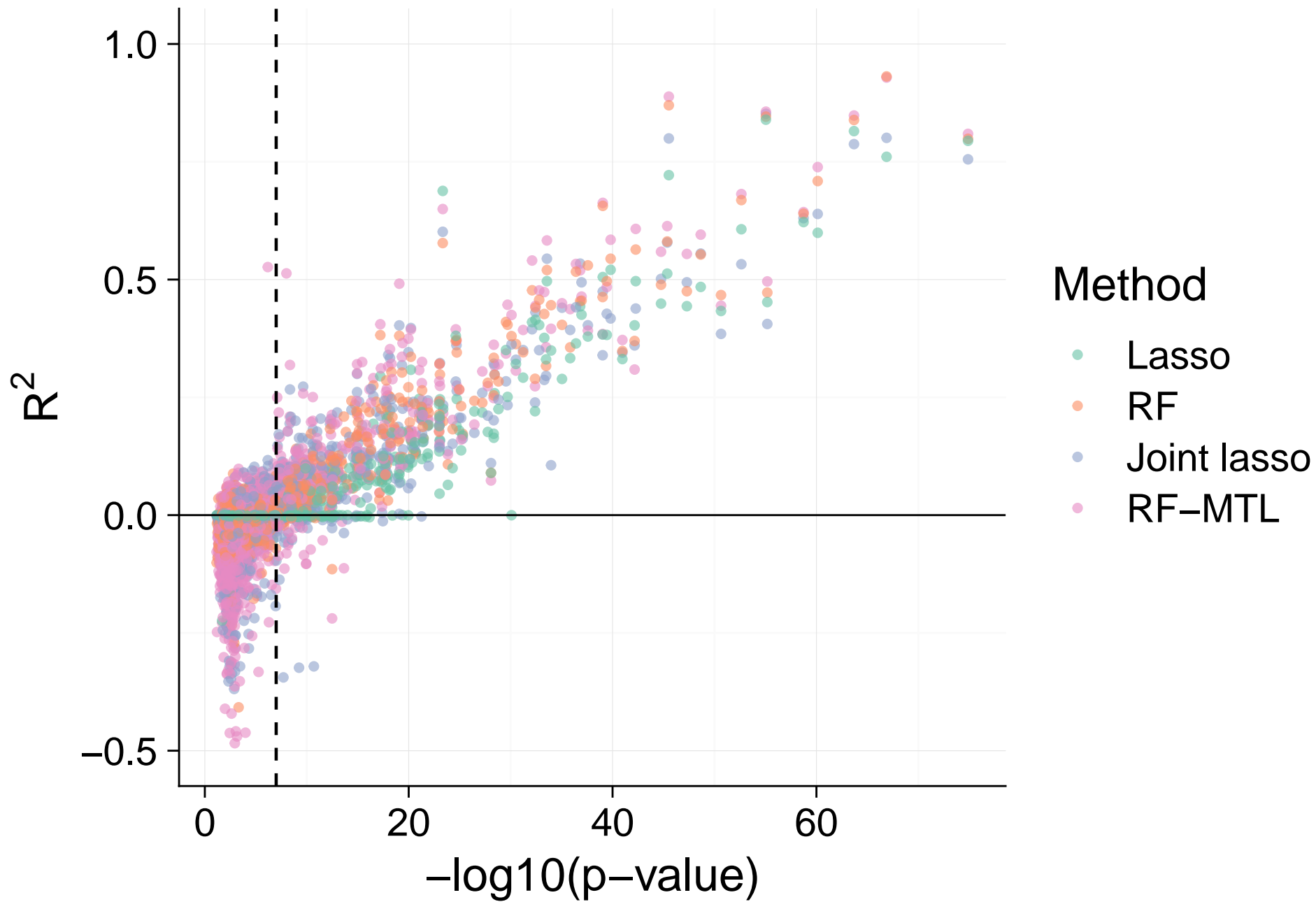

Supplement: Supplementary file 1 — Supporting information. [file GEPI-45-324-s006.pdf]

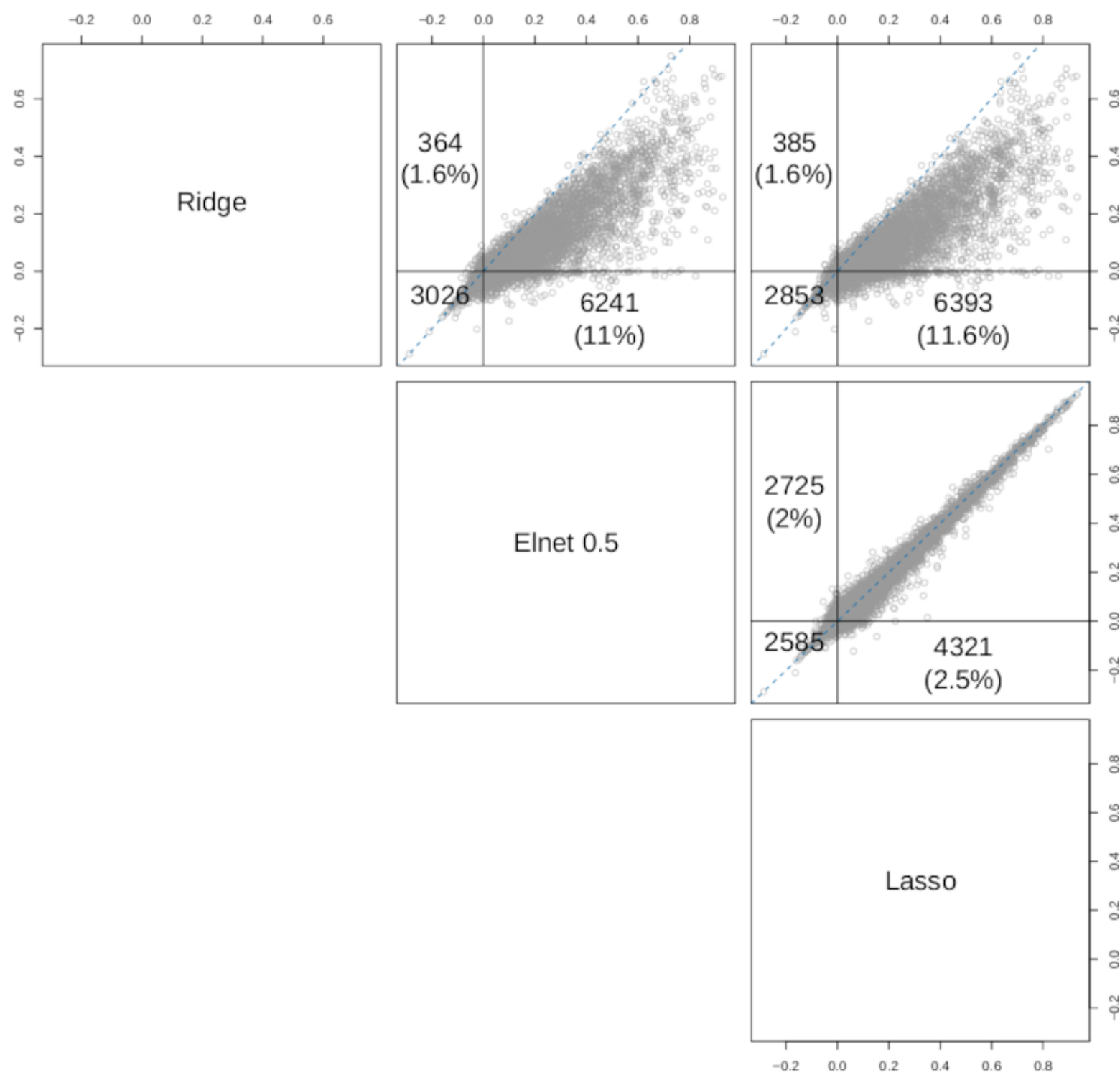

(a)

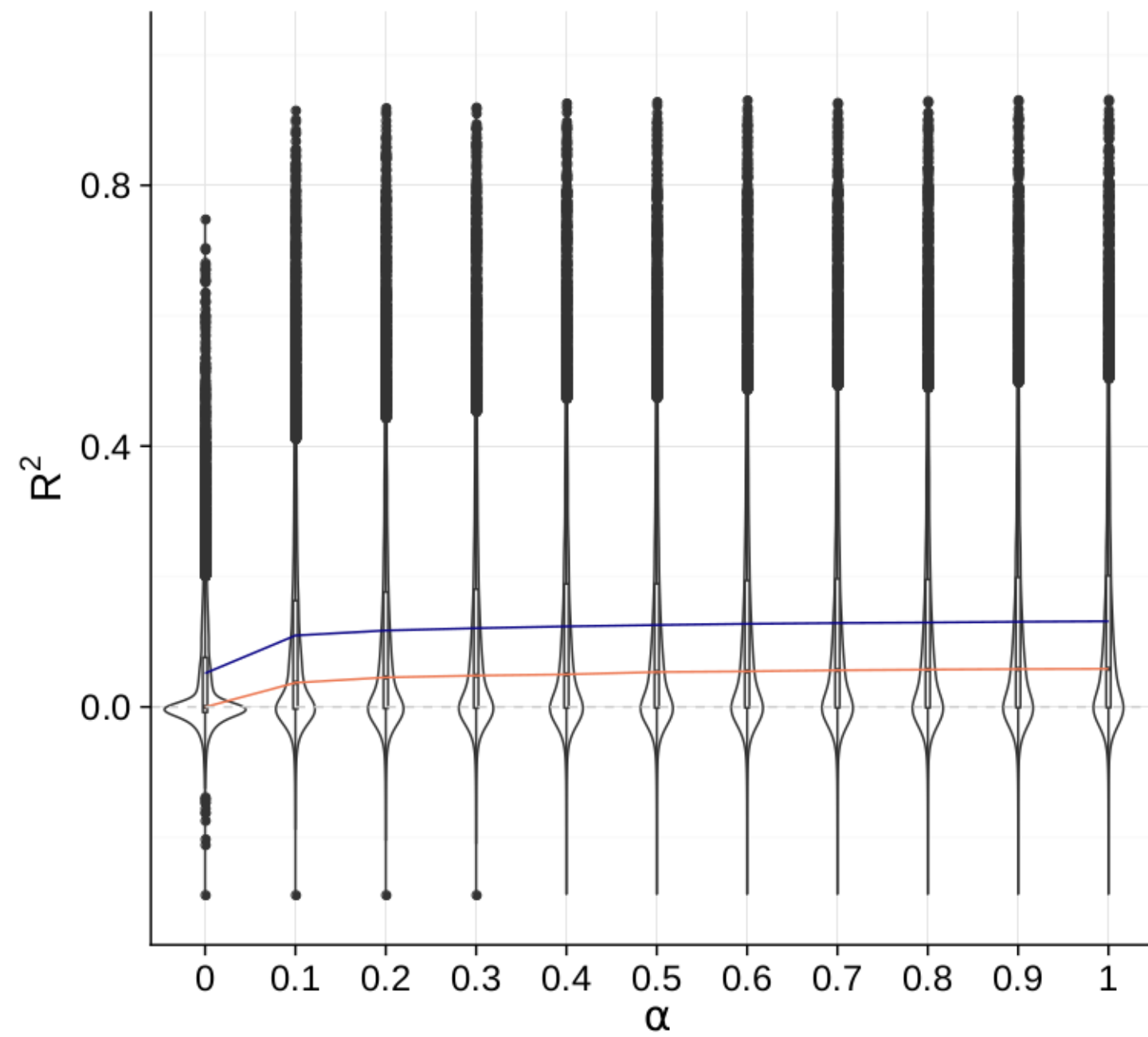

(b)

Supplement: Supplementary file 2 — Supporting information. [file GEPI-45-324-s007.pdf]

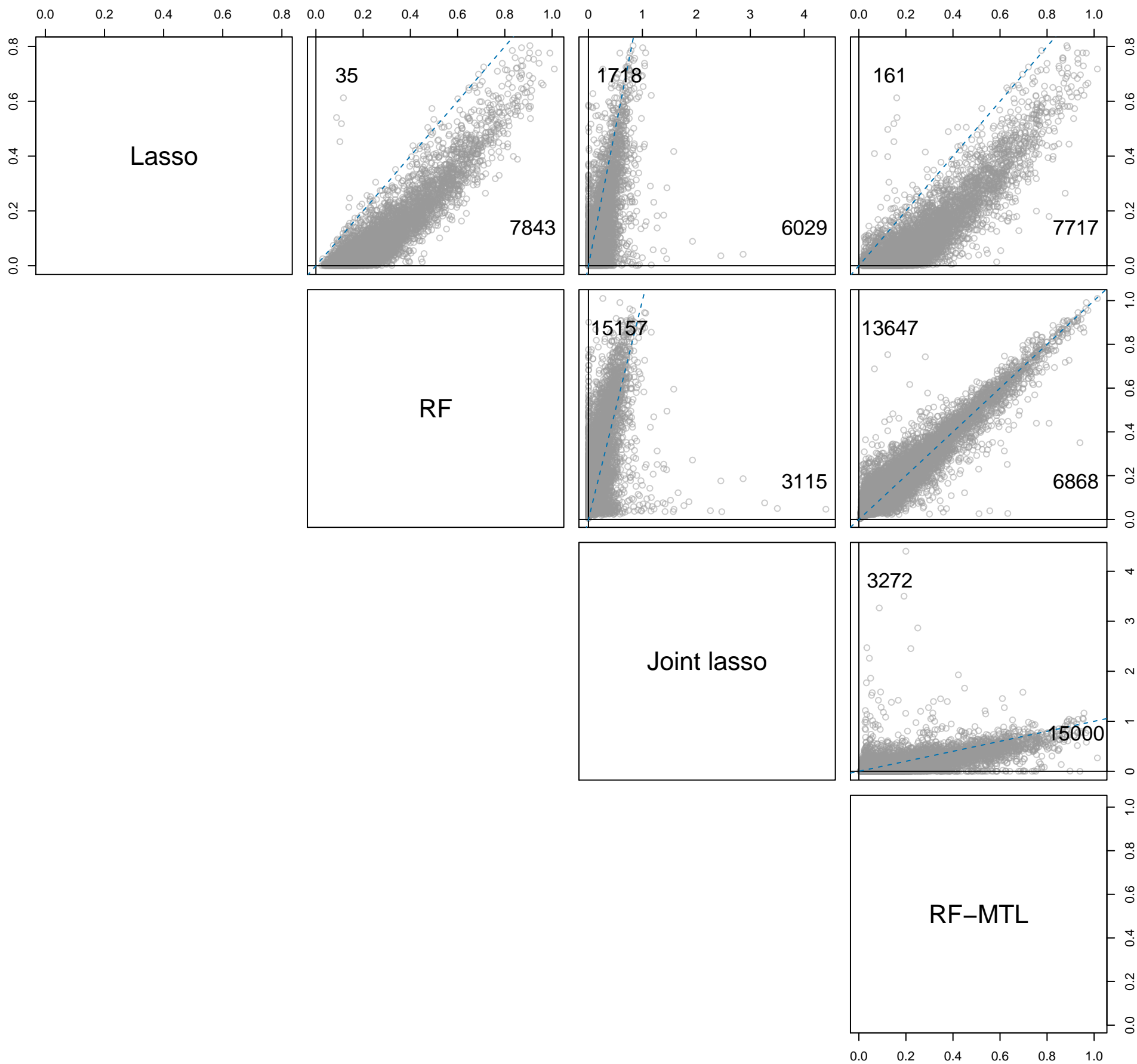

Supplement: Supplementary file 3 — Supporting information. [file GEPI-45-324-s010.pdf]

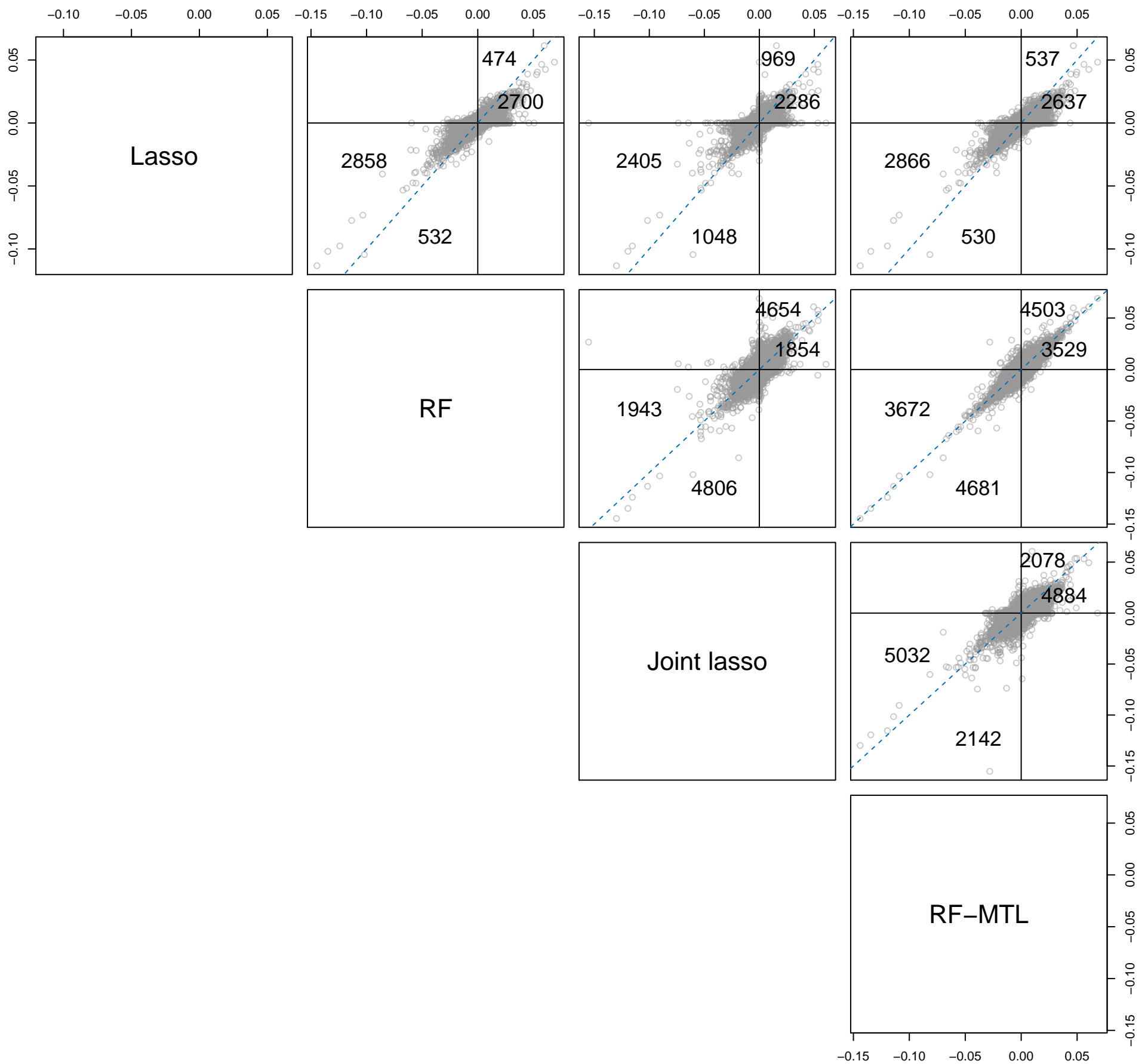

Supplement: Supplementary file 4 — Supporting information. [file GEPI-45-324-s001.pdf]

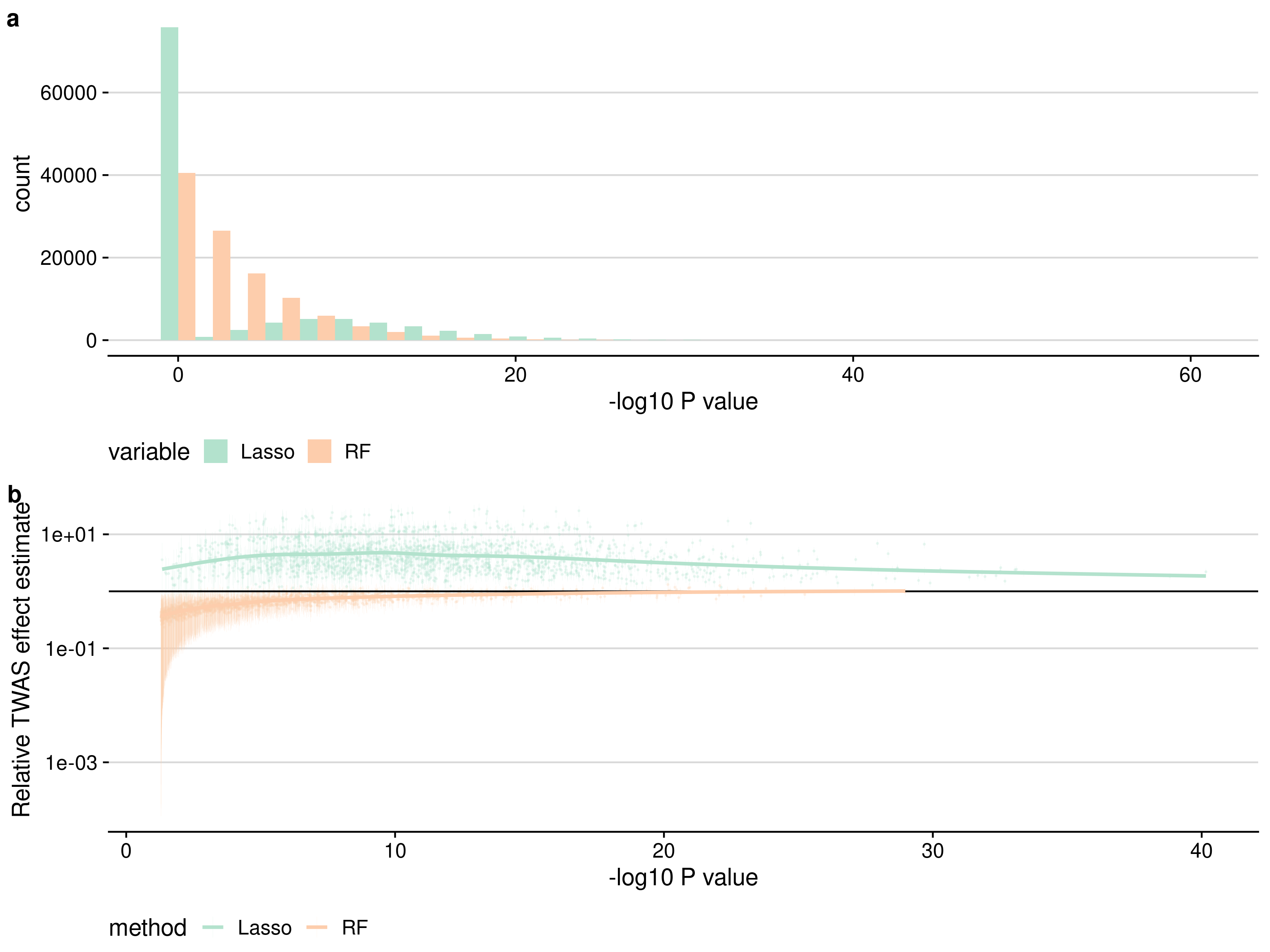

Supplement: Supplementary file 5 — Supporting information. [file GEPI-45-324-s003.png]

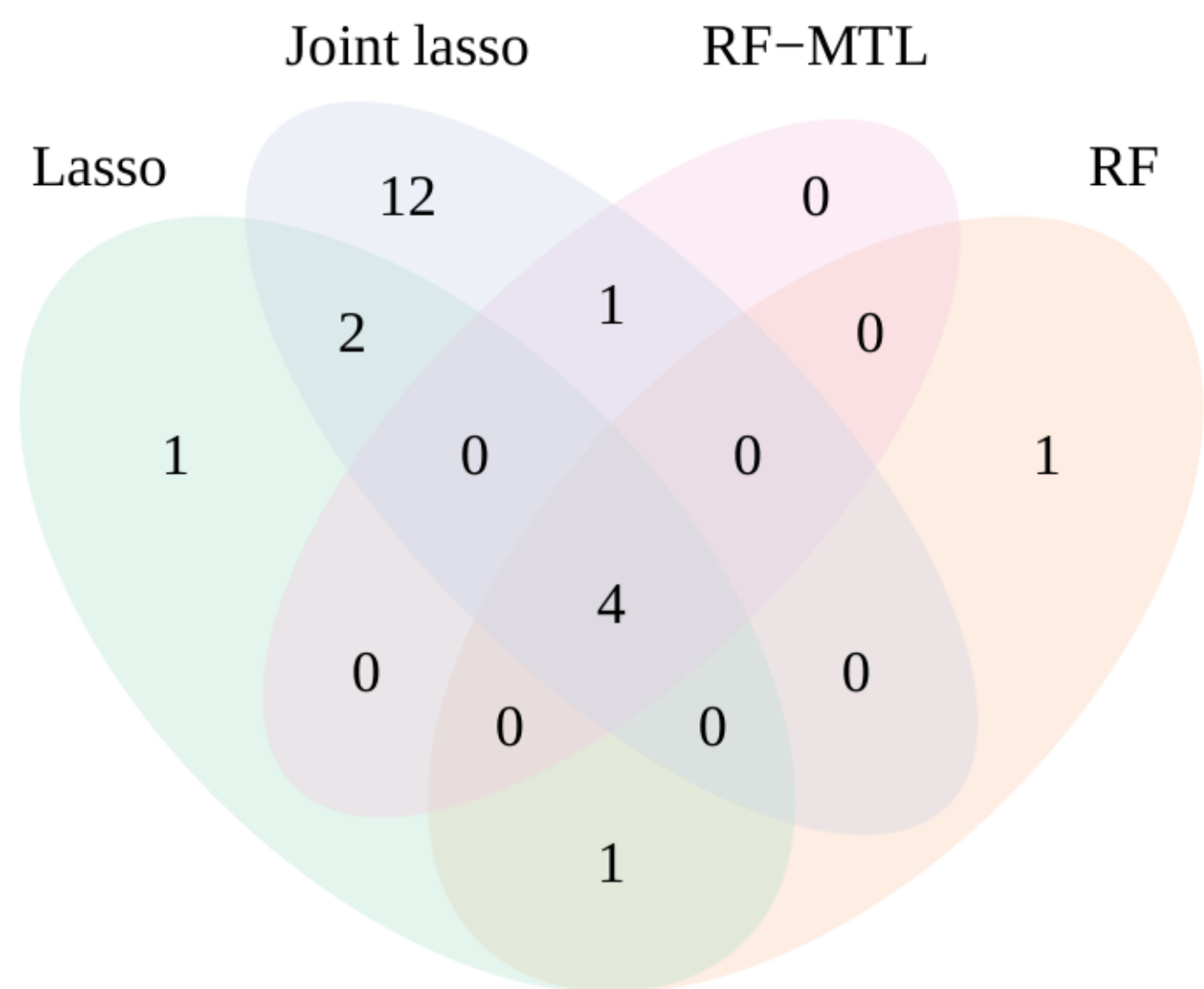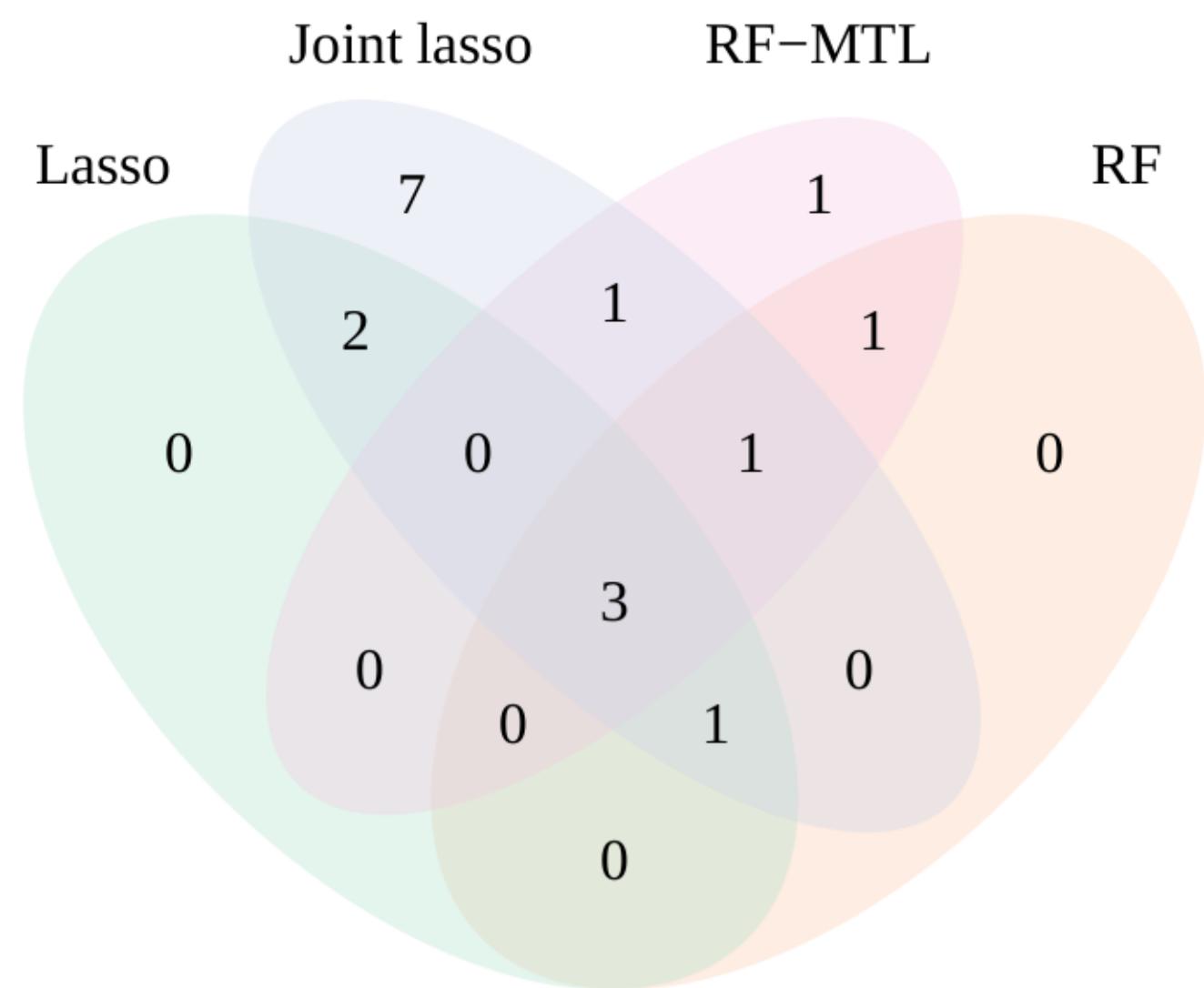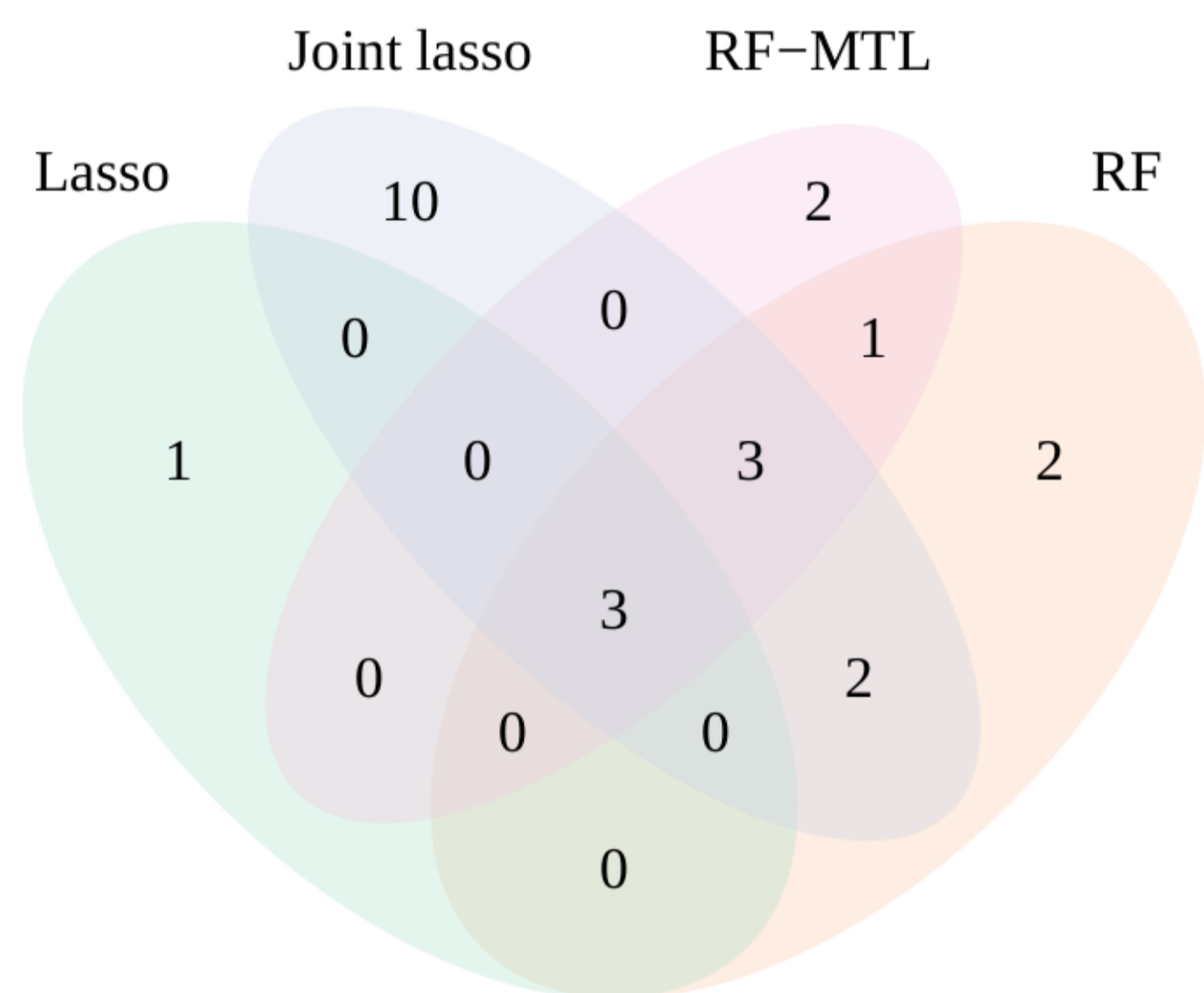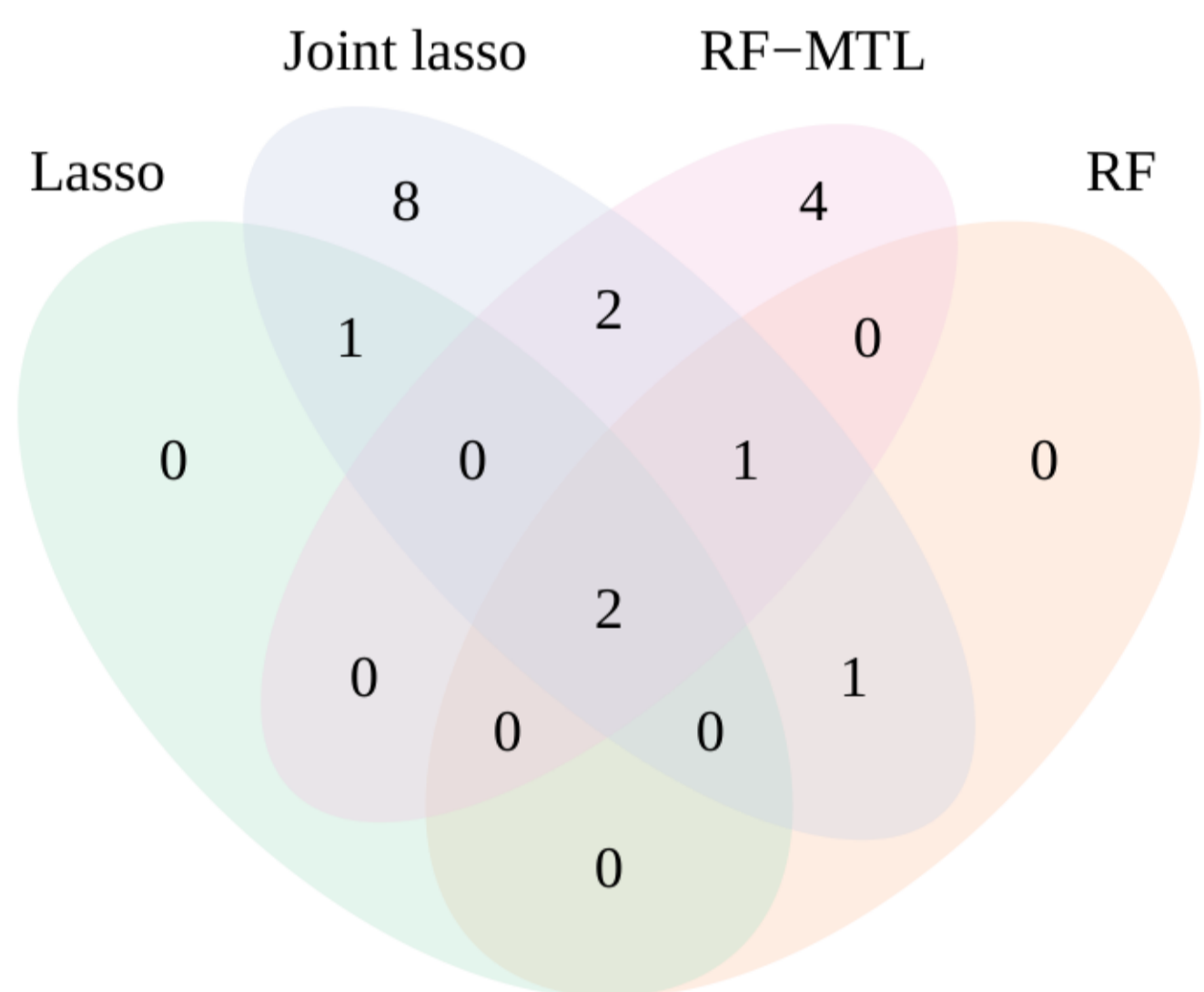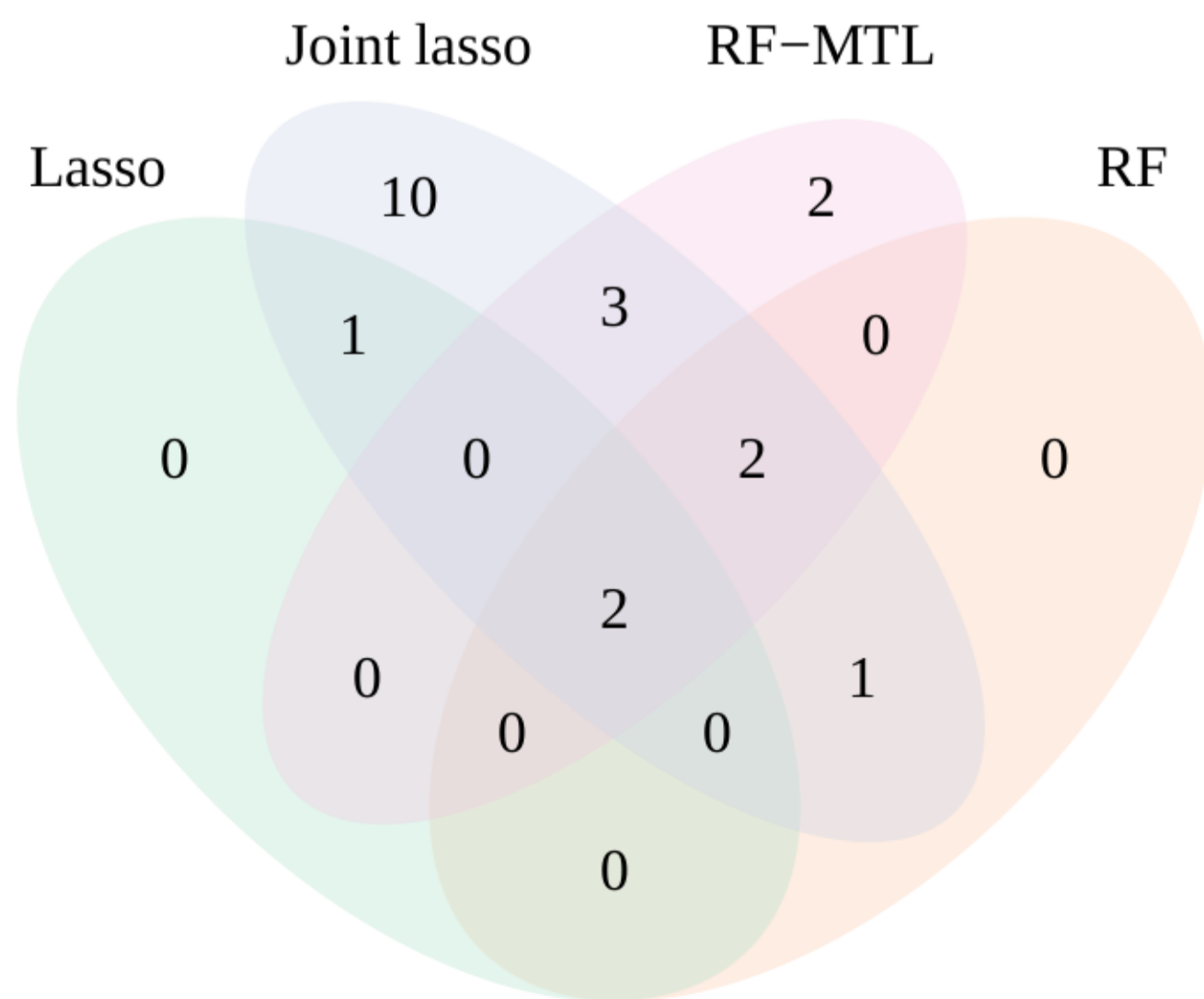

Supplement: Supplementary file 6 — Supporting information. [file GEPI-45-324-s008.pdf]

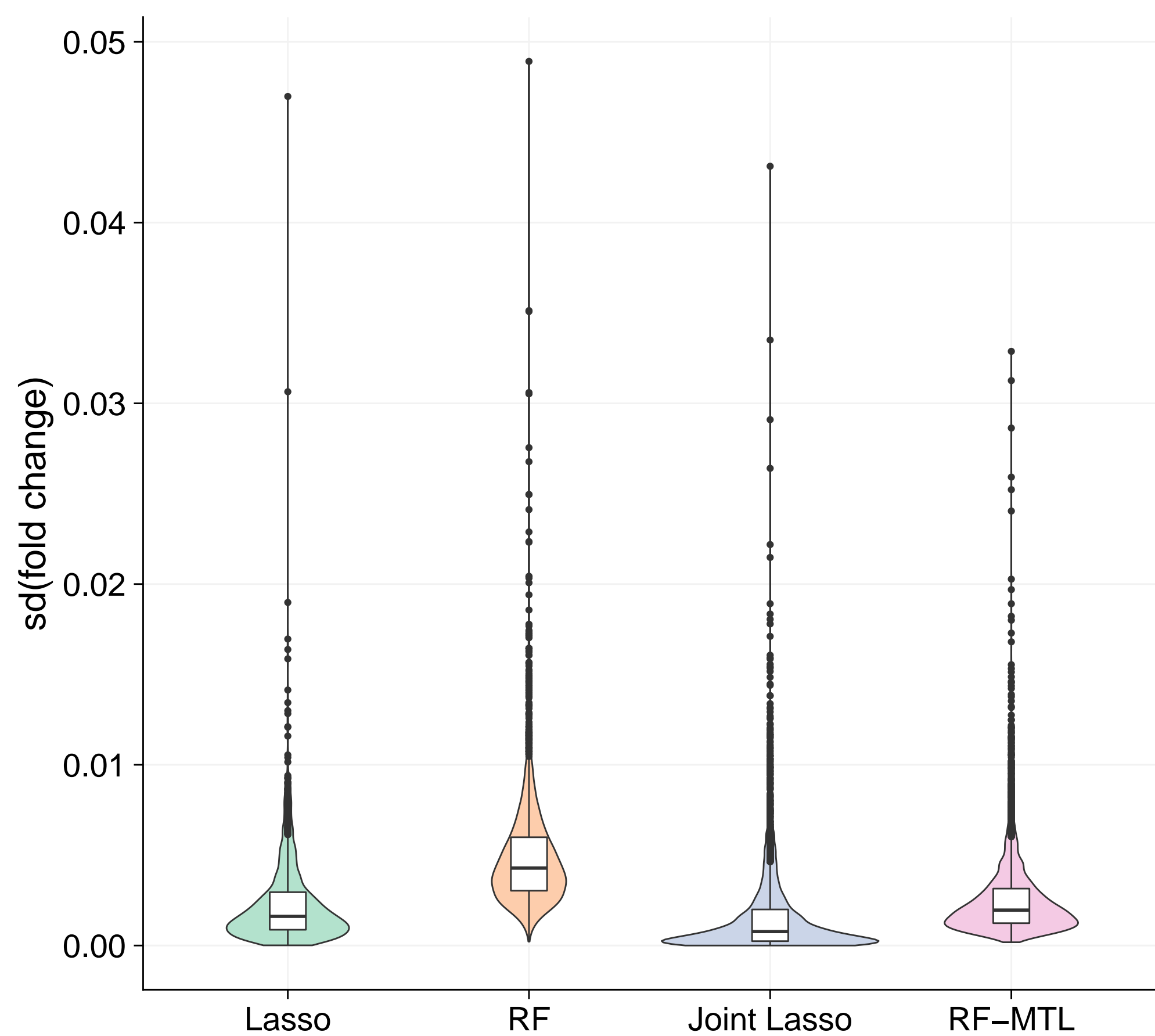

Supplement: Supplementary file 7 — Supporting information. [file GEPI-45-324-s004.pdf]
