# Supplementary material for: Multi‐tissue transcriptome‐wide association studies
Source: Genet Epidemiol. 2020 Dec 28;45(3):324–37. doi: 10.1002/gepi.22374 (PMC8048510; doi:10.1002/gepi.22374)
Supplement: Supplementary file 10 — Supporting information. [file GEPI-45-324-s005.docx]

**Table S1.** T1D data of (Barrett et al., 2009) comprising Wellcome Trust Case Control Consortium (WTCCC) (Burton et al., 2007) and Type 1 Diabetes Genetics Consortium (T1DGC) samples.

**Table S2.** Target Validation analysis of TWAS genes by method. The top 5 diseases ranked by relevance *p*-value, and the rank of four type 1 diabetes-related terms are shown.

**Figure S1.** Identifying a *p*-value threshold for the eQTL analysis. Performance of the four expression prediction methods, as assessed by $R^{2}$ on a test set, plotted against the minimum

*p*-value of the eligible (cis) SNPs for each probe/cell pair on chromosome 22 (3040 regressions for each method). The vertical dashed line is at $x=7$ (i.e. minimum *p*-value $=10^{-7}$).

**Figure S2.** (a) Pairwise comparison of performance ($R^{2}$on a 30% test set) of elastic net for $\alpha\in\{0,0.5,1\}$. Each point represents a probe-cell pair. Points above the red line show increased performance for the method to the left of each plot, while points below the red line show increased performance for the method underneath the plot. The three numbers represent, clockwise, starting top left: points with positive $R^{2}$ for the $x$-axis method above the $x=y$ line, points with positive $R^{2}$ for the $y$-axis method below the line, points with negative $R^{2}$ for both methods; average advantage in brackets. (b) Performance of elastic net for varying values of $\alpha$, evenly spaced between 0 and 1, on the eQTL dataset of Fairfax et al. ($R^{2}$ on a 30% test set). Note that the values 0 and 1 correspond to the ridge regression and lasso, accordingly. Each violin plot, with the embedded boxplot, aggregates all regressions for a given $\alpha$. The purple and orange lines are mean and median values of $R^{2}$, respectively.

**Figure S3.** Pairwise comparison of variance of imputed expression values for the four methods. The blue dashed line is the $x=y$ line. Numbers above and below the line correspond to the number of regressions for which the $y$-axis method has larger variance for the imputed predictions than the $x$-axis method and vice versa, respectively.

**Figure S4.** Pairwise comparison of predicted fold change for the four methods. The blue dotted line is the $x=y$ line. In the positive, quadrant the numbers above and below the line designate the number of regressions for which the $y$-axis has a larger predicted fold change than the $x$-axis method, and vice versa. Likewise for the numbers in the negative quadrant, except here the numbers relate to absolute fold change.

**Figure S5.** Effects of lasso regularisation on TWAS. **a** Lasso-TWAS *p*-values amongst simulations with shared eQTL/GWAS causal variants show a spike at *p*=1, and a longer tail than RF, indicating that weaker effects are missed by lasso, but that stronger effects can show greater significance compared to RF. **b** TWAS effect estimates (estimated causal effect of expression on GWAS trait) are underestimated for weak effects for RF, tending to 1 for stronger effects. For lasso, TWAS effect estimates are systematically over estimated, even for well-powered studies.

**Figure S6.** Venn diagrams showing unique SP-genes identified by the four methods, by cell type.

**Figure S7.** Violin plots (with inscribed boxplots) of standard deviations of predicted fold change for different cell types for each probe, per method. For each method, only probes with predictions for at least three cell types were considered.
